# Reverse Takotsubo Cardiomyopathy after Orthotopic Liver Transplantation. A Case Report

**DOI:** 10.2478/jccm-2022-0002

**Published:** 2022-05-12

**Authors:** Lucian Mihalcea, Isac Sebastian, Mihail Simion-Cotorogea, Artsiom Klimko, Gabriela Droc

**Affiliations:** 1 Department of Anesthesiology and Intensive Care I, Fundeni Clinical Institute, Bucharest, Romania; 2 Carol Davila University of Medicine and Pharmacy Faculty of Pharmacy, Bucharest, Romania; 3 Laboratory of Molecular Neuro-Oncology, Department of Neurology, University Hospital Zurich and University of Zurich, Zurich, Switzerland

**Keywords:** orthotopic liver transplantation, reverse Takotsubo, cardiogenic shock

## Abstract

**Introduction:**

Takotsubo cardiomyopathy is a rare reversible type of heart failure, often precipitated by emotional stress; other risk factors include intracranial bleeding, ischemic stroke, sepsis, major surgery, pheochromocytoma. The clinical, electrical and blood sample analysis features resemble those of a myocardial infarction- however, they occur in the absence of angiographic coronary filling defects.

**Case presentation:**

A 61-year-old male patient, 71 kg, 175 cm, underwent liver transplantation for Child-Pugh B cirrhosis secondary to mixed viral hepatitis (B and D). His medical records revealed mild mitral, aortic, and tricuspid insufficiencies and heart failure with preserved ejection fraction. An initially uneventful perioperative stage was succeeded by cardiogenic shock (cardiac index – 1.2 l/min/ sqm), which the patient developed 24 hours after the intervention. Elevated cardiac markers and ECG abnormalities showing ST-T changes in the V2-V5 leads were additionally noted. Transesophageal echocardiography (TEE) revealed an acute onset reduction in the left ventricular systolic function secondary to basal hypokinesia. No coronary obstruction was detected by percutaneous angiography. The above findings lead to the diagnosis of reverseTakotsubo cardiomyopathy. Further, the patient developed acute kidney injury and liver graft failure, succumbing within 48 hours after the surgical procedure.

**Conclusions:**

We report a rare case of reverse Takotsubo cardiomyopathy in a male patient after orthotopic liver transplant.

## Introduction

Stress cardiomyopathy or Takotsubo cardiomyopathy (TC) is a rare reversible type of heart failure, which occurs mostly in postmenopausal women, secondary to emotional stress [1].

The clinical presentation of stress cardiomyopathy is the same as that of an acute coronary syndrome: dyspnea, acute retrosternal chest pain, ECG changes, and cardiac enzyme abnormalities, with normal angiographic findings. Specific ultrasound findings usually reveal a left ventricle with poor regional contraction, manifested by end-systolic left ventricular apical ballooning.

Furthermore, because of its complex pathophysiology, this condition is likely underdiagnosed. The main pathophysiologic mechanisms of the disease are endogenous or exogenous catecholamine excess, leading to microvascular dysfunction and coronary artery spasm [2]. Experimental studies have shown that myocardial catecholamine toxicity could act as a negative inotrope by switching from Gs to Gi-protein coupled signaling, exhibiting a negative effect on the cardiac contractile function [3].

Nowadays, other factors seem to be involved in precipitating this condition as well, such as intracranial bleeding, head trauma, ischemic stroke, sepsis and septic shock, major surgery, overproduction of endogenous catecholamines (pheochromocytoma), or administration of exogenous catecholamines [4]. ACC/AHA recognize this condition as a unique form of reversible cardiomyopathy, which can last for months after the initial precipitating stress [5].

The aim of our study is to raise clinician awareness of this rare form of stress cardiomyopathy, reverse Takotsubo, in critically ill patients presenting with cardiogenic shock.

## Case presentation

**Fig. 1:**
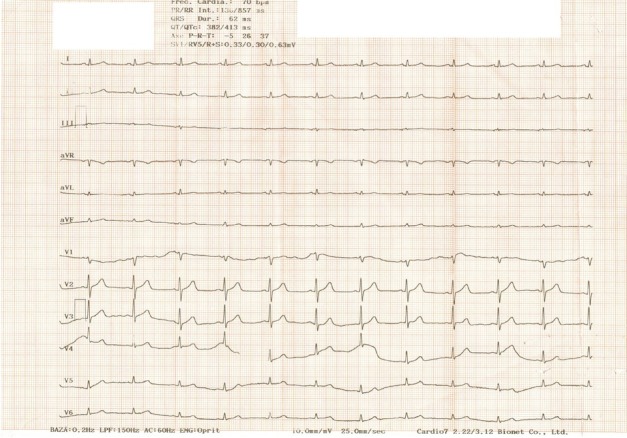
Preoperatively ECG showing flattened T waves in DIII, low voltage waves in limb leads

A 61-year-old male patient, 71 kg, 175 cm underwent orthotopic liver transplantation for Child-Pugh B liver cirrhosis secondary to mixed viral hepatitis (B and D). The patient had a sodium MELD score of 27 points. His medical records included mild mitral, aortic, and tricuspid insufficiencies and heart failure (NYHA II) with preserved left ventricle systolic function. Blood sample analysis revealed severe thrombocytopenia, moderate normochromic anemia, and a cirrhotic coagulopathy, with an INR of 2.1, an aPTT of 45 sec, fibrinogen levels of 153 mg/dl and a platelet count of 66.000/mm^3^. The ECG showed normal sinus rhythm, a normal QRS axis, flattened T waves in DIII, and diminished voltage in the limb leads (Fig.1). Structural causes of the preoperative ECG findings were investigated and ruled out via transthoracic ultrasonography (i.e., dilated cardiomyopathy, constrictive pericarditis, and pericardial effusion). Furthermore, history was negative for infiltrative systemic diseases or previous myocardial infarction. Biochemical results revealed mild hyponatremia, hyperbilirubinemia, normal heart enzymes and no inflammatory syndrome [6, 7]. The CT scan revealed a fibrotic structure of the liver and an umbilical hernia.

The patient underwent intravenous induction using 3 μg/kg fentanyl (Chiesi Pharmaceuticals GmbH, Austria),1.5 mg/kg propofol (Fresenius Kabi GmbH, Austria) and 1 mg/kg succinylcholine (Takeda Austria GmbH). General anesthesia was maintained with sevoflurane (Abbvie Deutschland GmbH & Co., Germany) (1.7%-2% end expiratory gas concentration), fentanyl (0.5-1.5 μg/kg/h) and rocuronium (N.V. Organon, Holland) (0.6 mg/kg initial dose, followed by 0.2 mg/kg every 45-60 minutes). The following parameters were monitored: intermittent blood pressure measurement, continuous-mediated five channel ECG, continuous pulse oximetry, breathing rate, end tidal CO2, end tidal sevoflurane, lung minute-volume, and lung pressures.

After anesthesia induction, the left radial artery was cannulated for invasive blood pressure monitoring – a central venous catheter was placed in the right jugular vein. Extensive hemodynamic monitoring was performed by way of the PiCCO thermodilution technique, using the central venous catheter for cold fluid injection and the temperature sensor in the right femoral artery. Per the manufacturer’s guidelines, 20ml ice cold saline boluses were used for PiCCO calibrations [8].

During the procedure, the patient received potassium-depleted crystalloid infusions and human albumin for intravascular volume resuscitation. For hemostatic correction, a total of 2g fibrinogen, 1g tranexamic acid, 4 thrombocyte concentrate units, 6 erythrocyte concentrate units, and 2 fresh frozen plasma units were administered, in accordance with thromboelastometric findings. Hemodynamic changes were countered in accordance with PiCCO monitoring, using norepinephrine infusion (AS Kalceks, Latvia), in doses ranging from 0.04-1.27μg/kg/min (Table 1). Overall, the ascites volume was estimated at 8000 ml, and bleeding volume at 3000 ml. Lateral clamping time was 17 minutes and the anhepatic phase lasted for 53 minutes. The hepatic artery resistance index was 0.71, with a portal vein velocity of 35cm/s. For immunosuppression, the patient received 500mg methylprednisolone (Pfizer Manufacturing Belgium N.V, Belgium) and 20mg basiliximab (Novartis Farmacéutica S.A., Spain) intraoperatively. Additionally, a dose of 10.000 IU human hepatitis B immunoglobulin was administered, in accordance with the local protocol. Postoperatively, the intubated, hemodynamically stable, and residually sedated patient was admitted to the ICU.

**Table 1:** Intraoperative hemodynamic parameters. CVP- central venous pressure, CI-cardiac index, SVRI- systemic vascular resistance index, ELWI-extra lung water index, SVV- stroke volume variation, Norepi- norepinephrine, BP- blood pressure.

Time since induction (min)	0	30	60 *	90	120	150**	180	210	240	270	300	330	360
CVP (mmHg)	14	13	9	8	8	16	15	14	15	14	13	13	14
CI (l/min/m^2^)	2.9	2.8	2.5	2.5	2.4	2.6	2.7	2.8	2.7	2.7	2.7	2.6	2.6
SVRI (dynes·sec/cm^5^/m^2^)	1954	1901	1870	1656	1402	1103	1302	1520	1660	1596	1584	1767	1989
ELWI (ml/kg)	8.9	9.2	10.1	10.1	11	11	10.9	10.6	10.7	10.6	10.8	10.2	10.3
SVV (%)	12	13	13	12	10	18	18	14	14	15	14	13	12
Norepi (mcg/kg/min)	0.04	0.12	0.14	0.36	0.6	1.27	0.82	0.68	0.3	0.33	0.33	0.25	0.15
BP (mmHg)	105/65	100/60	100/63	90/60	90/60	115/70	110/70	115/70	120/70	110/70	105/60	110/65	115/70

*clamping time, **declamping time

**Fig. 2:**
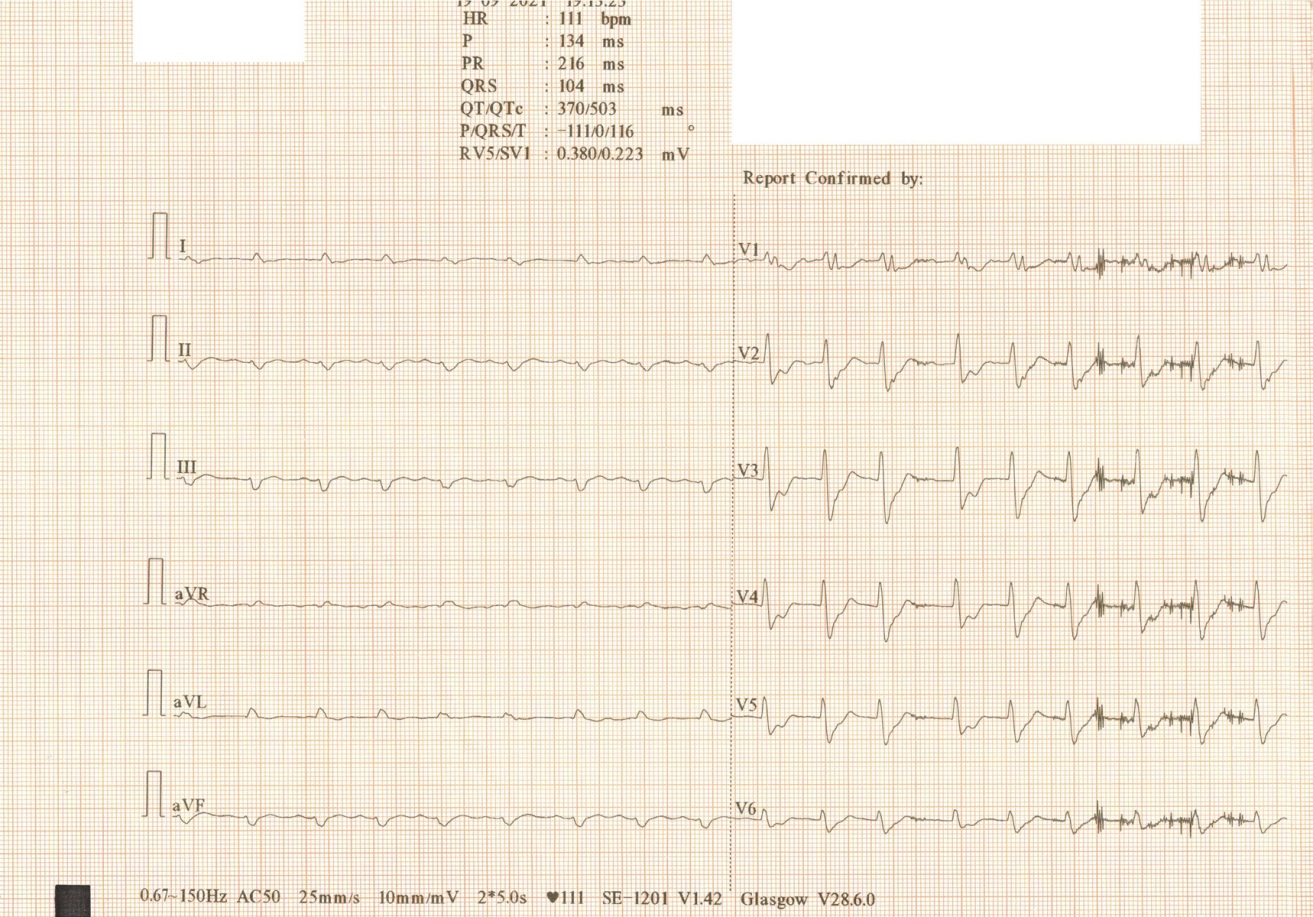
Postoperatively ECG tracing revealing ST-T abnormality in V2-V5 leads.

Twenty-four hours after surgery, the patient developed cardiogenic shock, detected using PiCCO measurements (CI=1.2 l/min/sqm, SVRI=3800 dynes-sec/ cm^–5^/m^2^), requiring prompt inotropic and concomitant vasopressor therapy. Continuous infusion with dobutamine (Solupharm Pharmazeutische Erzeugnisse GmbH, Germany) with a starting dose of 2.5 μg/kg/min was initiated along with norepinephrine, which was reintroduced at a starting dose of 0.3 μg/kg/min. The maximum hemodynamic support therapy flow rates were 10.7 μg/kg/min dobutamine and 2.4 μg/kg/min norepinephrine, respectively (Table 2). The cardiac enzyme assay revealed a marked increase in hs-cTnI (295 times over the cut-off value), NT-proBNP (150 times over the cut off value), and CK, with the preponderance of CK-MB (11 times over the cut-off value). ECG showed new ST-T abnormalities in V2-V5 leads, which were not present in the preoperative ECG. (Fig.2)

**Table 2:** Postoperative hemodynamic parameters.

Postoperative hours	+4	+12	+20	+24	+28	+32	+36	+40	+44	+48
CVP (mmHg)	14	16	15	21	22	19	18	18	18	18
CI (l/min/m^2^)	2.7	2.6	2.4	1.4	1.32	1.34	1.52	1.5	1.55	1.66
SVRI (dynes·sec/cm5/m2)	1790	1800	1900	2700	3505	3670	3400	3160	3250	2950
ELWI (ml/kg)	9.3	8.5	7.9	11.5	13.3	12	14	12	11	10.3
SVV (%)	14	10	11	12	12	13	13	13	12	12
Norepi (mcg/kg/min)	0.1	-	-	0.3	1.82	2.08	2.08	2.2	2.4	2.4
Dobutamine (mcg/kg/min)	-	-	-	2.5	5.3	8.33	10.7	10.7	10.7	10.7
BP (mmHg)	112/87	108/83	109/79	103/73	101/71	83/53	92/52	103/61	107/66	99/61

CVP- central venous pressure, CI- cardiac index, SVRI- systemic vascular resistance index, ELWI-extra lung water index, Norepi- norepinephrine, BP- blood pressure

**Fig. 3:**
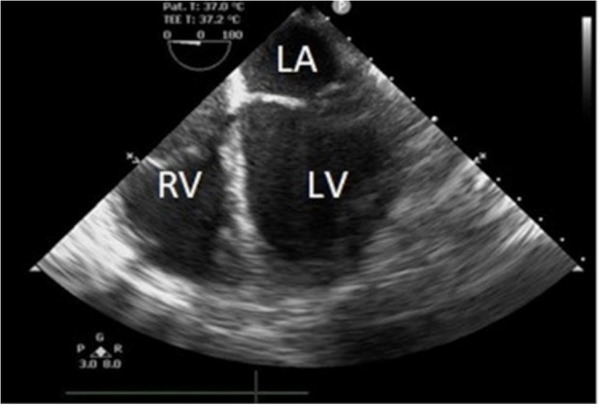
TEE imaging showing hypokinesia of the LV base. No RV motion anomalies were identified.

**Fig. 4:**
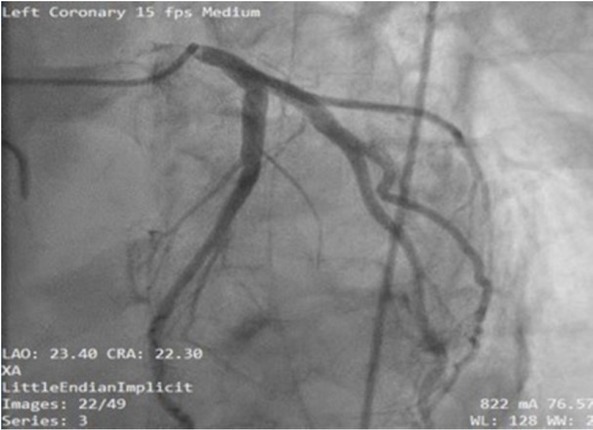
Angiography showing no coronary occlusion

Due to improper TTE windows, a TEE was performed, revealing an acute reduction in the systolic function localized to the base of the left ventricle. The EF was estimated at 35%with mild mitral, aortic, and tricuspid insufficiency. The right ventricle and the apex had normal contractile function (Fig.3). No coronary occlusion was identified during coronary angiography (Fig.4).

**Fig. 5:**
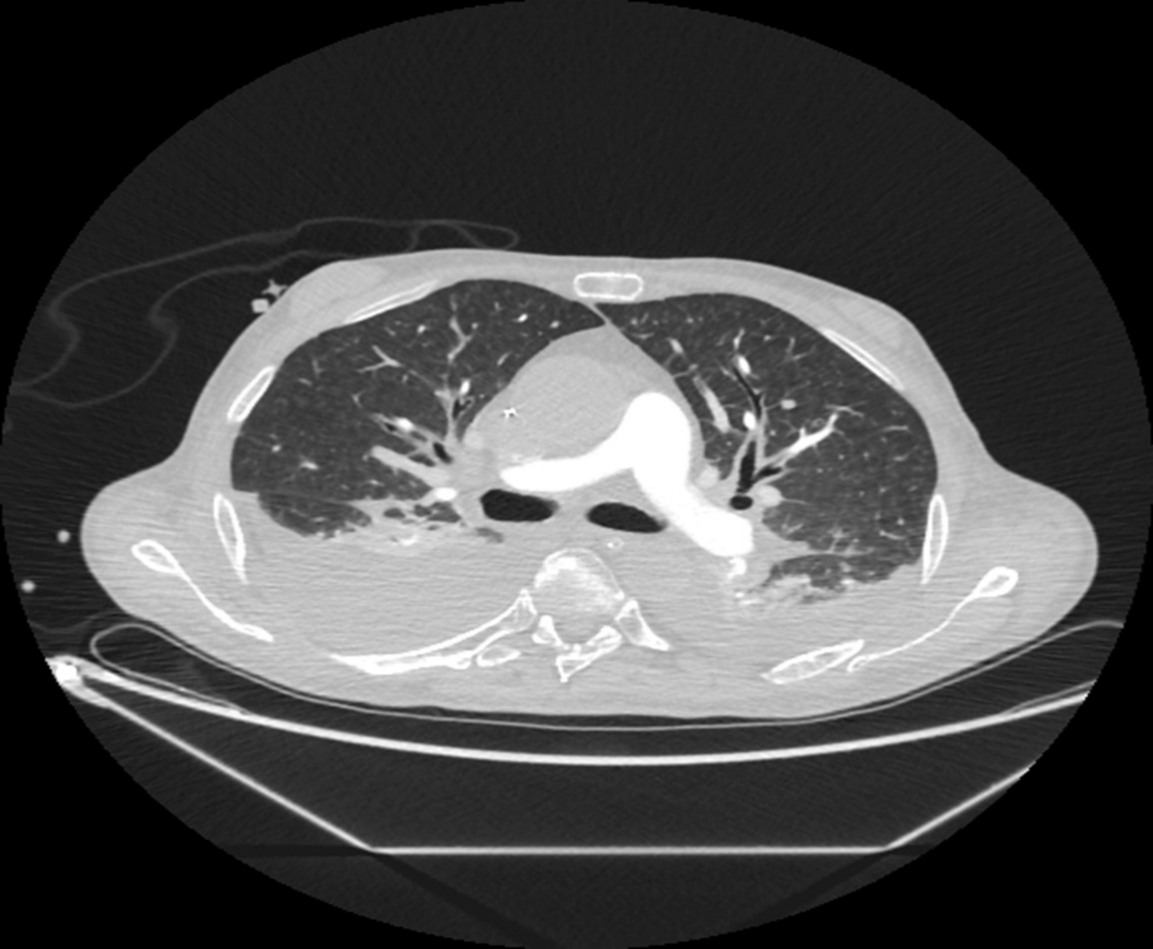
Postcontrast thoracic CT-scan revealing bilateral pleural effusions. No signs for central thromboembolism or pneumothorax were documented.

We considered the following as differential diagnoses of shock: hypovolemia, pulmonary embolism, pneumothorax, and septic shock. The postoperative PiCCO measurements and TEE ruled out hypovolemia (Table 2). Pulmonary embolism and pneumothorax were excluded through a thoracic CT-scan (Fig.5). Postoperative bacteriological screening revealed no pathogen and sepsis blood markers (PCT and presepsin) were inconclusive. Moreover, hemodynamic parameters pointed to cardiogenic shock (Table 2).

Considering the association between the hemodynamic parameters, cardiac enzyme elevation, ECG changes, ultrasound findings, and absence of coronary obstruction pointed towards the diagnosis of reverse Takotsubo cardiomyopathy was reached. Further imagistic and urinary findings excluded the presence of pheochromocytoma. Evidence-based therapy for stress cardiomyopathy was applied.

Considering the intricate postoperative condition of the patient, no anticoagulant therapy was introduced, because of the inappropriate liver graft synthesis capacity with subsequent coagulopathy: an increase of INR (6.3 times over the cut-off value), APTT (1.8 times over the cut-off value), and decrease in fibrinogen (50% lower than the cut-off value) and platelet count (75% lower than the cut-off value). Secondary to persistent low cardiac output syndrome, the patient developed acute kidney injury (AKIN stage 3), with an increase in serum creatinine of 0.4 mg/dl and a urine output less than 0.3 ml/kg/h over 24 hours, which was managed using CVVHDF. Consequently, no ACE inhibitors were given. Only low dose beta-blocker therapy was administered, due to the major hemodynamic instability of this patient. Further, the patient developed acute liver graft failure and died 48 hours postoperatively.

## Discussion

The modified Mayo Clinic criteria for the diagnosis of Takotsubo cardiomyopathy are: transient contractility abnormalities in the left ventricular mid segments with or without apical involvement; regional wall motion abnormalities that extend beyond a single epicardial vascular distribution; the absence of obstructive coronary disease or angiographic evidence of acute plaque rupture; new ECG abnormalities (ST-segment elevation and/or T-wave inversion) or modest elevation in cardiac troponins; and the absence of pheochromocytoma or myocarditis [9].

Angiography revealed no abnormalities in our patient, in accordance with the modified Mayo criteria [9]. Ghadri et al. developed the InterTak score in 2017, which identified female gender as the most important risk factor for Takotsubo cardiomyopathy. Despite this, our patient was male, demonstrating the importance of knowledge of this disease, regardless of patient’s gender [10].

Regarding wall motion abnormalities, there are several types of stress cardiomyopathy: 1) apical type – the typical form of this disorder presenting with depressed mid and apical segment contraction (compensatory hyperkinesis of the basal walls could often be observed; this type was present in 81.7% of patients in the International Takotsubo Registry study); 2) mid-ventricular type; 3) basal type; 4) focal type; 5) global type [11]. Our patient was identified with basal type stress cardiomyopathy or *reverse Takotsubo*, which is a rare form [12].

According to Stiermayer et al., only 10% of the patients develop cardiogenic shock [13]. The data supports the acute onset cardiogenic shock identified postoperatively in our patient.

Regarding ECG changes, our report is in accordance with the literature. Frangieh et al. reported a high sensitivity of non-ST elevation changes in stress cardiomyopathy [14]. In other studies that examined stress cardiomyopathy, 7.9% of patients were initially misdiagnosed with non-ST elevation acute coronary syndrome [15].

Pelliccia et al. reported that cardiovascular comorbidities could predispose to stress cardiomyopathy, with the most important being dyslipidemia, obesity, and hypertension [16]. Another recent article revealed that male gender is associated with worse outcomes [17]. No other specific risk factors, like chronic heart failure or valvulopathies, were identified [17].

In our patient, cardiogenic shock, although rare, would have been more likely to occur during surgery, secondary to reperfusion syndrome [18]. reverse Takotsubo cardiomyopathy was diagnosed based on acute onset postoperative cardiogenic shock in the presence of cardiac enzyme abnormalities, ECG changes, and lack of significant coronary angiographic lesions. TEE studies revealed hypokinesis of the ventricular base with no abnormalities on angiography. No clinical signs like dyspnea or retrosternal chest pain were documented.

## Conclusions

We illustrated a rare form of Takotsubo cardiomyopathy (reverse Takotsubo cardiomyopathy) in a patient who underwent orthotopic liver transplantation in a tertiary, university-affiliated national centre of liver transplantation and surgery, with atypical ECG findings and high levels of cardiac enzymes, explained in part by the surgical procedure and secondary kidney failure. [19, 20].
